# Comparisons of readmissions and mortality based on post-discharge ambulatory follow-up services received by stroke patients discharged home: a register-based study

**DOI:** 10.1186/s12913-018-3809-z

**Published:** 2019-01-05

**Authors:** Jayson O. Swanson, Tron Anders Moger

**Affiliations:** Department of Health Economics and Health Management, Institute of Health and Society, University of Oslo, PO Box 1089, Blindern, NO-0317 Oslo, Norway

**Keywords:** Stroke, Patient readmission, Mortality, Follow-up studies, Ambulatory care, Home nursing, Patient discharge

## Abstract

**Background:**

Few studies have focused on post-discharge ambulatory care for stroke patients and subsequent differences in readmission and mortality rates. Identifying groups at higher risk according to services received is important when planning post-discharge follow-up in ambulatory care. According to a recent Whitepaper by the Norwegian Government, patients receiving ambulatory care should have follow-up with a general practitioner (GP) within 14 days of hospital discharge.

**Methods:**

All home discharged stroke cases occurring in Oslo from 2009 to 2014 were included. 90- and 365-day all-cause readmissions and mortality were compared separately for patients categorized based on services received (no services, home nursing, ambulatory rehabilitation and home nursing with ambulatory rehabilitation) and early GP follow-up within 14 days following discharge. Variables used to adjust for differences in health status and demographics at admission included inpatient days and comorbidities the year prior to admission, calendar year, sex, age, income, education and functional score. Cox regression reporting hazard ratios (HR) was used.

**Results:**

There were no significant differences in readmission rates for early GP follow-up. Patients receiving home nursing and/or rehabilitation had higher unadjusted 90- and 365-day readmission rates than those without services (HR from 1.87 to 2.63 depending on analysis, *p* < 0.001), but the 90-day differences disappeared after risk adjustment, except for patients receiving only rehabilitation. There were no significant differences in mortality rates according to GP follow-up after risk adjustment. Patients receiving rehabilitation had higher mortality than those without services, even after adjustment (HR from 2.20 to 2.69, *p* < 0.001), whereas the mortality of patients receiving only home nursing did not differ from those without services.

**Conclusions:**

Our results indicate that the observed differences in unadjusted readmission and mortality rates according to GP follow-up and home nursing were largely due to differences in health status at admission, likely unrelated to the stroke. On the other hand, mortality for patients receiving ambulatory rehabilitation was twice as high compared to those without, even after adjustment and irrespective of also receiving home nursing. Hence, assessing the needs of these patients during discharge planning and providing careful follow-up after discharge seems important.

**Electronic supplementary material:**

The online version of this article (10.1186/s12913-018-3809-z) contains supplementary material, which is available to authorized users.

## Background

Stroke is a leading cause of mortality and disability globally with many patients receiving rehabilitation services [1, 2]. These patients are sensitive to readmissions [3–6] and have high mortality rates [7], making the post-discharge period crucial. Coordination and vertical integration of care among primary and secondary (specialist) care is a persistent issue challenging many healthcare systems worldwide [8, 9]. Trends such as shorter hospital lengths of stay (LOS), expanded outpatient care and reducing hospital beds have led to the need for increasing more cost-effective ambulatory follow-up options such as home nursing and ambulatory rehabilitation services to substitute more extensive inpatient treatments. To ensure this, appropriate coordination between care levels, often referred to as transitional care, is imperative [8, 10, 11].

Literature on demographic and clinical risk factors measured before and during initial hospitalization and their effect on outcomes such as readmissions and mortality for stroke patients is abundant [3, 4, 12–17]. However, very few studies use population-based data to compare outcomes related to patients’ use of ambulatory services after discharge. Some focus on specific interventions in randomized trials [18, 19], some are based on small samples [20] and others only study one service type [21]. Patients receiving early follow-up visits by a general practitioner (GP) [22] or home nursing care after discharge could have significantly different readmission and mortality rates than those without such services. Receiving these services could imply relatively poorer post-discharge health, either attributable to the stroke itself or to health status before admission, leading to significantly higher observed outcome rates. On the other hand, closer patient follow-up and more attentive care resulting from receiving these services could attenuate any difference in outcomes. Patients requiring additional ambulatory rehabilitation could be expected to have higher rates compared to those without, due to service provision directly dependent upon post-stroke health. Post-discharge rehabilitation prescribed for patients with rehabilitation requirements exceeding that received during the hospital stay also likely characterizes different levels of assistive needs than what necessitates home nursing. The magnitude of these differences is uncertain and identifying groups at higher risk of adverse outcomes is important for follow-up in ambulatory care and informative for both primary and secondary healthcare providers.

Norwegian general and university hospitals are owned by the central state and administered by four geographically distributed regional health authorities. Reimbursement from the central state is based on activity reported to the register by hospitals. Primary care including GPs, ambulatory rehabilitation and home nursing are organized and managed by local municipal governments. Integration, coordination and communication between care providers at different levels are important for discharge planning and follow-up and have been documented affecting post-discharge outcomes [23–25]. All Oslo hospitals (three local and one university) have stroke units. If a hospital believes a patient will need home nursing or rehabilitation (at home and health centers) from the municipality, prior to discharge it notifies the office of health and care services in the borough the patient resides. The borough then takes appropriate action, and the patient should, in principle, receive any needed services directly after discharge. A recent Whitepaper by the Norwegian Government stated all patients receiving ambulatory services post-discharge should have GP follow-up within 14 days [26].

Most studies are typically constrained to specific population segments or demographics due to data availability and source limitations. As a first of its kind, this study allowed us to uniquely employ data linked from multiple registers covering an entire population, Oslo. Collaboration with Oslo Municipal Health Administration made constructing complete individual health service utilization histories possible. This includes hospitalization, primary care and ambulatory services such as home nursing, long-term care (LTC) and rehabilitation before and after hospitalization for stroke, as well as readmissions and mortality up to 1 year post-discharge. In doing so, we describe the post-discharge services utilized by stroke patients discharged home. The main aim is to compare outcomes for stroke patients with early follow-up by healthcare workers at different levels, in this case, general practitioners (GPs), home nursing and ambulatory rehabilitation, compared to not receiving such services. Specifically, we consider 90- and 365-day all-cause readmissions and mortality.

First, we descriptively compare health status, demographics, service use and outcomes for patients receiving standalone or combinations of ambulatory care services (rehabilitation, home nursing and GP follow-up) within 14 days of being discharged home to those without services. Second, we compare outcomes both unadjusted and adjusted for health status and demographics at admission. Notable reductions in unadjusted differences in outcomes after adjustment could indicate that these differences are due to factors not related to the stroke itself, but rather to health status and demographics at admission. Separate analyses are performed for GP care services and GP follow-up.

## Methods

### Defining the sample

Stroke was defined using International Classification of Diseases 10th revision (ICD-10) codes I60-subarachnoid haemorrhage, I61-intracerebral haemorrhage, I63-cerebral infarction and I64-stroke, not specified as haemorrhage or infarction. Patients residing in Oslo and hospitalized from 2009 to 2014 with a primary stroke diagnosis were identified from the Norwegian national inpatient register (Norsk Pasientregister). The register has been deemed to be complete and accurate, particularly for stroke diagnoses validity [27, 28], hence identification of all relevant patients is expected. Patients admitted for stroke during the 365 days before index admission were excluded from analyses to focus on incident cases. All-cause readmissions occurring within 365 days post-discharge were identified, and patients with in-hospital mortality were excluded.

### Data

The inpatient register provided additional information used for describing the sample and risk adjustment. We calculated total non-stroke related inpatient days and identified comorbidities from primary and secondary diagnosis ICD-10 codes, 365 days prior to index admission. The identified comorbidities are a list of 14 conditions ranging from hypertension, depression and dementia to cancer and coronary artery disease that has been validated elsewhere [29]. Age at admission, LOS and sex were also collected. Patient income the year before admission, the highest education level attained and an indicator of disability pension received since 1992 were collected from Statistics Norway. Death date was acquired from the national cause of death register (DÅR, Dødsårsaksregisteret) and GP visits were identified from the national general practitioner reimbursement register (KUHR, Kontroll og Utbetaling av HelseRefusjon). Activities of daily living (ADL) scores valid 30 days prior to admission were identified from the Oslo municipal health and care service register (Oslo kommunes fagsystem for omsorgstjenestene), as well as any home nursing services, ambulatory rehabilitation or stays in long-term care before and after hospitalization. All municipal service variables and ADL scores included start and end dates. We constructed ADL sum-scores from 17 measurements the municipality uses to evaluate patients’ service needs (eight physical ADL items, seven instrumental ADL items, vision and hearing) where higher values indicate greater need. Individual items scored as “not relevant” were coded as zero when calculating sum-scores. Considering that even minor services (e.g., safety alarms) require an ADL evaluation, patients without ADL evaluation were assumed not needing municipal ambulatory services and also coded as zero. We constructed variables using inpatient and municipal data indicating services patients received each day from discharge to 1 year after. Various data examination processes, reporting incentives and coding and control systems function to ensure high levels of validity and accuracy in these registers [30, 31], and unique individual national identification numbers widely used in Norwegian administrative and healthcare registers enabled deterministic linkage [32, 33].

### Statistical methods

**Fig. 1:**
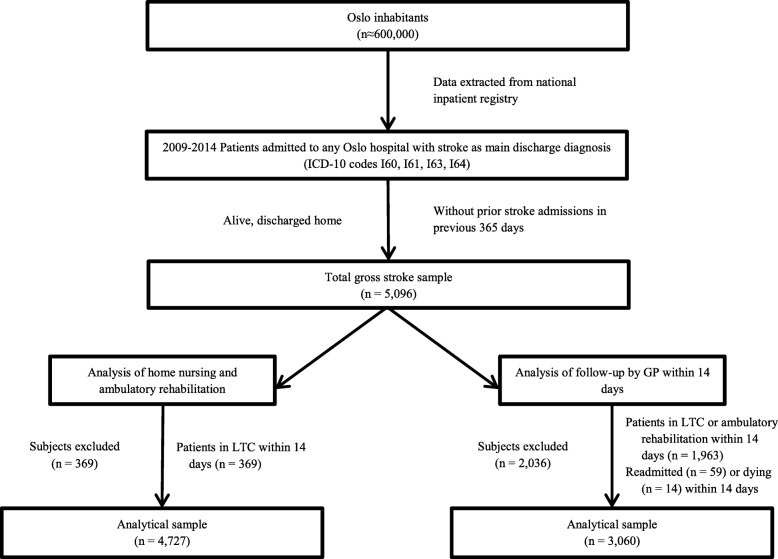
Flowchart summary of analytical samples with exclusion criteria

For descriptive analysis, we divided patients into four care categories based on ambulatory services received during the first 14 days post-discharge prior to any readmissions. The reference category was defined as patients discharged home without services. Assuming the municipality can quickly and accurately determine patients’ needs with effective post-discharge planning, this category should have the lowest readmissions and mortality rates. The other three categories received standalone home nursing or rehabilitation, or a combination of both. Additionally, separate analyses were performed comparing patients without GP follow-up visits within 14 days to those with at least one visit. Patients receiving ambulatory rehabilitation within 14 days were excluded from the GP follow-up analyses, as these patients often have GP contacts included in the service which are not visible in the data. To standardize the period used for identification of post-discharge visits patients readmitted or dying within 14 days post-discharge were also excluded. Thus, the periods analyzed for GP follow-up were 14–90 days and 14–365 days. Statistics are presented as means and interquartile ranges (IQR) for continuous variables and as percentages for categorical variables. Differences between groups were tested using Kruskal-Wallis and Mann-Whitney tests for continuous variables and chi-square tests for categorical variables. Construction of the analytical sample, including inclusion and exclusion criteria, is summarized in Fig. 1.

Cox regression analyses were applied to four different outcomes (90- and 365-day readmission and 90- and 365-day mortality) to compare short- and long-term crude and adjusted rates estimating hazard ratios (HR) with 95% confidence intervals (95% CI). Adjusted models account for calendar year, sex, age, income, education, ADL score, total inpatient days the year before admission, and eight of the 14 comorbidities having pre-admission prevalence rates > 0.5% (listed in Additional file 1: Table S1 and Additional file 2: Table S2). Calendar year was included as a yearly indicator. Age was categorized from < 50 years to > 89 years with intervals every 5 years in-between. Inpatient days the previous year was categorized into 0 days, 1–10 days and > 10 days. ADL sum-score was categorized into 0, 1–24, 25–39 and ≥ 40. Income and education were categorized according to Tables 1 and 2. Observations with missing values for education (< 2%) were excluded from the regressions.

**Table 1:** Descriptive statistics for patients discharged to four care categories

	No services (*n* = 2605)	Rehab (*n* = 689)	Home nursing (*n* = 535)	Nursing with rehab (*n* = 898)	
Variable	Mean, IQR (%)				*p*-value
ICD-10,%					
I60	4.0	2.4	2.8	1.0	< 0.001
I61	8.8	16.8	9.3	11.5	
I63	83.4	77.9	82.6	83.4	
I64	3.8	2.9	5.3	4.1	
LOS stroke	12.2 (9)	21.6 (19)	14.5 (13)	16.4 (13)	< 0.001
Male,%	59.8	44.4	40.9	35.4	< 0.001
Age	66.5 (19)	80.0 (14)	78.3 (16)	83.1 (11)	< 0.001
LOS previous year	0.4 (0)	0.7 (0)	1.8 (0)	2.0 (0)	< 0.001
Comorbidities	0.2 (0)	0.2 (0)	0.4 (1)	0.5 (1)	< 0.001
% with ADL score > 0	10.4	44.6	69.0	78.4	
ADL score	2.6 (0)	12.5 (24)	20.9 (32)	26.9 (19)	< 0.001
Disability pension,%	24.9	30.9	39.6	27.7	< 0.001
Income,^c^%					
< €20,000	18.8	23.8	27.8	24.3	< 0.001
€20,000-31,000	21.8	32.9	34.8	37.3	
€31,000-41,000	21.5	24.4	23.4	22.3	
> €41,000	37.9	18.9	14.0	16.1	
Education,%					
Primary	23.8	33.0	37.0	35.3	< 0.001
Secondary	42.0	43.5	43.6	46.4	
Tertiary	34.2	23.5	19.4	18.3	
Within 14 days,%					
GP visit	44.2	4.1	3.4	0.8	< 0.001
Within 365 days,^a^%					
Long-term care	1.3	34.3	8.7	35.5	< 0.001
Readmitted	12.3	26.4	24.5	25.6	< 0.001
Dead	5.9	22.7	12.2	29.3	< 0.001
Within 90 days,^b^%					
Readmitted	6.5	11.8	11.2	10.4	< 0.001
Dead	1.8	8.0	3.7	11.8	< 0.001

*LOS* Length of stay, *IQR* Interquartile range for continuous variables

^a^Data period 01.01.2009–31.12.2013

^b^Data period 01.01.2009–30.09.2014

^c^€1 = NOK9.75

**Table 2:** Descriptive statistics for patients with and without GP visits within 14 days post-discharge

	No GP within 14 days (*n* = 1899)	GP within 14 days (*n* = 1161)	
Variable	Mean, IQR (%)		*p*-value
ICD-10,%			
I60	3.6	4.2	0.15
I61	9.6	7.3	
I63	82.8	84.4	
I64	4.0	4.1	
LOS stroke	14.3 (12)	9.7 (7)	< 0.001
Male,%	55.8	57.4	0.39
Age	69.3 (20)	67.1 (20)	< 0.001
LOS previous year	0.6 (0)	0.4 (0)	0.22
Comorbidities	0.2 (0)	0.2 (0)	0.18
% with ADL score > 0	25.7	11.2	< 0.001
ADL score	7.3 (17)	2.7 (0)	< 0.001
Disability pension,%	26.9	27.9	0.55
Income,^c^%			
< €20,000	21.7	17.6	< 0.01
€20,000-31,000	24.3	23.6	
€31,000-41,000	21.8	21.8	
> €41,000	32.2	37.0	
Education,%			
Primary	25.1	25.2	0.95
Secondary	40.9	41.2	
Tertiary	30.6	31.7	
Within 365 days,^a^%			
Home nursing	37.1	10.0	< 0.001
Within 14–365 days,^a^%			
Long-term care	3.2	1.0	< 0.001
Readmitted	13.2	11.8	0.30
Dead	7.6	4.5	< 0.01
Within 14–90 days,^b^%			
Readmitted	5.6	5.0	0.49
Dead	1.8	1.3	0.29

*LOS* Length of stay, *GP* General practitioner, *IQR* Interquartile range for continuous variables

^a^Data period 01.01.2009–31.12.2013

^b^Data period 01.01.2009–30.09.2014

^c^€1 = NOK9.75

Patients may receive home nursing prior to the index admission and then switch to not receiving it post-discharge and vice versa. To check the stability of differences according to care categories, we performed sensitivity analyses (1) by analyzing only those patients receiving home nursing prior to the index admission and (2) by excluding patients with changes in home nursing status within 90 days post-discharge. Stroke cases occurring more than 1 year apart for the same patient causes duplicate identification in the data so robust standard errors were used. Non-proportional hazards in the Cox models were checked with plots of Schoenfeld residuals vs. time for each covariate. Data were analyzed using Stata version 14.2.

## Results

**Fig. 2:**
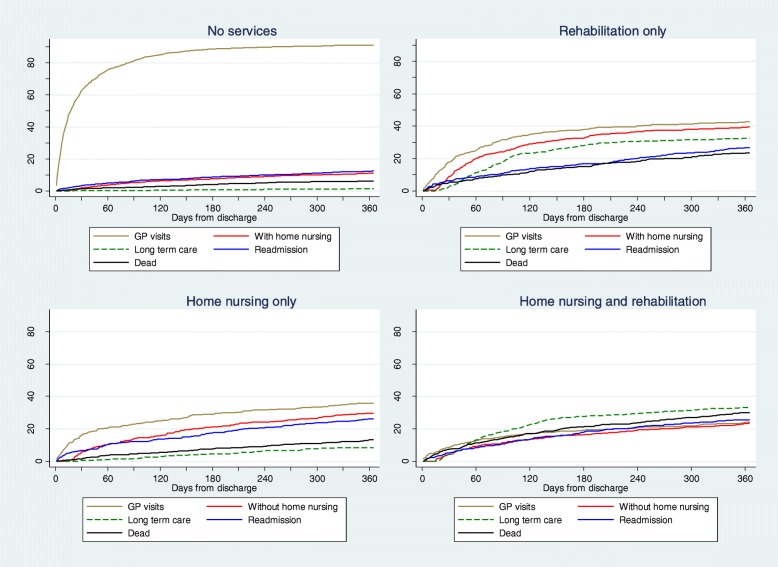
Cumulative proportions of patients in each state 1 year following discharge differentiated by initial services

Two thousand six hundred five patients (55%) were discharged without services, 689 (15%) with rehabilitation only, 535 (11%) with home nursing only and 898 (19%) with both home nursing and rehabilitation. 70% of patients discharged with home nursing also received the service prior to hospitalization consisting of 57% of patients in the home nursing only category and 75% in the home nursing and rehabilitation category. Less than 2% of those discharged without home nursing had received the service prior to admission. Across all care categories, 4% or less received rehabilitation at any point during the month prior to the index admission. Table 1 shows the descriptive results comparing the post-discharge care categories. Patients receiving services after discharge were generally frailer than those without, in terms of being older, having higher ADL scores, more inpatient days both prior to and during the stroke admission and receiving LTC between 14 and 365 days after discharge. These patients also had higher post-discharge readmission and mortality rates. Few patients discharged with services had GP follow-up within 14 days. Figure 2 graphically presents the cumulative service use and death status from discharge to 1 year after.

Table 2 presents descriptive results of patients with and without GP follow-up within 14 days after discharge. Patients without a GP visit were older and had longer stroke admission LOS, higher ADL scores, lower income and more frequent use of home nursing the year following discharge. Outcome rates were only significantly different for 365-day mortality.

Table 3 presents results of the Cox regression analyses (hazard ratios for all variables provided in Additional file 1: Table S1 and Additional file 2: Table S2). In the unadjusted post-discharge care category analyses, all categories indicated higher readmission rates compared to being discharged home without services. After adjustment for health status and demographics at admission, all differences in 365-day readmission rates were reduced but remained significant, whereas for 90-day readmissions only the difference for patients receiving standalone rehabilitation maintained significance. Those with standalone rehabilitation also had significantly higher 90- and 365-day adjusted rates than patients receiving home nursing with rehabilitation. All care categories had higher rates than the reference in the unadjusted mortality analyses. These results persisted in the adjusted models for both categories with rehabilitation, but not for patients receiving home nursing only. Furthermore, patients receiving home nursing with rehabilitation had higher rates compared to patients receiving standalone rehabilitation in the unadjusted analyses, but these also disappeared after adjusting for health status and demographics at admission. Hazard rates with and without GP follow-up were not significantly different in any analyses except for 14–365-day mortality, where the difference disappeared after risk adjustment.

**Table 3:** Cox regression hazard ratios with 95% confidence intervals for readmissions and mortality

	No services	Rehab	Home nursing	Home nursing with rehab	No GP visits	Visit to GP
Readmission						
90-day unadjusted	1	2.11	1.94	1.87	1	0.88
		1.61–2.76	1.43–2.62	1.44–2.42		0.63–1.24
90-day adjusted	1	1.45^*^	1.08	0.95	1	1.11
		1.06–1.97	0.74–1.57	0.67–1.34		0.75–1.64
365-day unadjusted	1	2.60	2.26	2.63	1	0.90
		2.15–3.13	1.84–2.77	2.21–3.13		0.73–1.11
365-day adjusted	1	1.80^*^	1.31	1.46	1	1.20
		1.45–2.22	1.02–1.66	1.16–1.84		0.95–1.51
Mortality						
90-day unadjusted	1	4.87^*^	1.88^*^	6.91	1	0.68
		3.38–7.01	1.12–3.14	4.98–9.57		0.38–1.22
90-day adjusted	1	2.69	0.81^*^	2.54	1	0.77
		1.80–4.03	0.45–1.47	1.70–3.81		0.42–1.42
365-day unadjusted	1	4.58^*^	2.24^*^	6.05	1	0.61
		3.62–5.78	1.65–3.02	4.90–7.47		0.44–0.85
365-day adjusted	1	2.38	1.00^*^	2.20	1	0.77
		1.83–3.09	0.71–1.41	1.68–2.89		0.54–1.09

^*^Significant difference to home nursing with rehabilitation, *p* < 0.05

Figure 2 indicates some patients switch from receiving home nursing to not, and vice versa, beyond 14 days post-discharge. At 90 days, these groups accounted for 10–20% of patients and removing them from the analyses as a sensitivity check did not alter the conclusions. Also, excluding the 30% of new home nursing recipients from the analyses did not change the regression results.

## Discussion

We observed higher readmission and mortality rates for stroke patients receiving home nursing and higher 365-day mortality for those without early GP follow-up. However, after adjusting for admission health status and demographic characteristics, the short-term readmission and all mortality differences disappeared and the long-term readmission difference was reduced. Hence, once a stroke patient is discharged and receives home nursing or lacks early GP follow-up, it seems important to consider his/her overall condition, based on factors like those we have included in the risk adjustment, before generalizing and flagging him/her for higher short-term readmission or mortality risk. The results further indicate that most of the unadjusted differences in readmissions and mortality for these groups of patients are attributable to pre-stroke factors, not the stroke itself. Like the long-term readmission difference for patients with home nursing, patients receiving rehabilitation continued to exhibit significantly higher rates of readmission and mortality than patients discharged without services even after risk adjustment.

ADL score, often unavailable in population-based studies, had the largest impact in reducing differences in outcomes between patients discharged without services and those receiving home nursing only. A related study with limited sample size did not find effects of functional dependence before stroke on post-discharge mortality [14]. With ADL sum-scores set to zero for patients without a pre-stroke evaluation, there is potential for underestimation of these scores, particularly in the reference category (only 10% having ADL-scores above zero). This would invalidate the conclusion that unadjusted differences in readmissions and mortality rates are due to pre-stroke factors. However, with the reference category patients more than 10 years younger (Table 1) than the other groups, and being discharged *without* services, this seems unlikely. Additionally, from a health provider’s perspective, patients without ADL-scores are regarded as lacking a need for services, making the approach used for employing this adjuster relevant.

Significantly higher readmission and mortality rates before and after risk adjustment for patients receiving ambulatory rehabilitation can be expected if one assumes only more severe cases receive ambulatory rehabilitation after discharge in addition to rehabilitation given in stroke units at hospitals. It was particularly striking that patients receiving only ambulatory rehabilitation had considerably longer stroke LOS than the other categories. Significant differences in the ICD-10 stroke diagnoses between care categories were the basis for not including these variables in the risk adjustment. Due to data limitations, gauging or quantifying stroke severity with measures (e.g. the National Institutes of Health Stroke Scale (NIHSS)) commonly employed in administrative data based studies [34], was not possible. At the same time, stroke LOS and ICD-10 code are not pre-stroke factors and have been argued as unreliable for use in adjustment to account for differences in stroke severity [34], making interpretations of rate differences difficult. The higher risk-adjusted readmission rates for patients receiving only rehabilitation compared to patients receiving both home nursing and rehabilitation could be due to needs for services not assessed or captured by the municipality (indicated by low percentage of patients having ADL sum-score above zero), greater stroke severity (indicated by longer stroke LOS) or better post-discharge follow-up for patients with home nursing. Including additional service needs indicators for risk adjustment, especially directly quantifiable measures of stroke severity, is important in future studies.

So few standalone home nursing recipients having GP follow-up within 14 days was unexpected, but this could be due to communication and coordination between GPs and home nurses not visible in the data. Low GP follow-up compliance after stroke has been found elsewhere with additional associations. Patients with greater age, pre-stroke ADL dependency, and prior stroke were less likely to receive doctor’s follow-up [21], and earlier outpatient follow-up could likely prevent most avoidable readmissions [22]. Another study initially conducting unadjusted analyses found patients discharged home without healthcare more likely to be readmitted, which challenges our findings, but after adjustment in multivariate analyses the associations disappeared to agree with our analyses [35]. Also contradicting our findings, outpatient department follow-up rates after initial stroke hospitalization were found to be positively associated with readmission and mortality risk, but it was noted, study subjects’ health status could limit adverse outcome preventability [36]. The same study also found lower likelihood of negative outcomes for patients who received inpatient/outpatient rehabilitation, but this difference could be affected by including variables for inpatient rehabilitation, which we did not [36]. Preventive effects of early GP or ambulatory follow-up on outcomes such as readmissions and mortality are plausible based on the aforementioned studies, but difficult to ascertain in our study due to lack of stroke severity information.

Excluding deaths and readmissions within 14 days when comparing hazard rates for early GP follow-up was done to ensure uniformity in the time periods utilized to identify GP visits for all patients. Patients readmitted or dying within the first few days post-discharge have less opportunity for GP follow-up visits, thus including these patients when comparing rates is potentially misleading. This was a major reason why no differences were observed in the GP analyses excluding these patients. As a supplement to Table 2, including events within 14 days yielded significant rate differences e.g., 90-day readmission rates of 8.4% (no GP) and 5.5% (GP, *p* < 0.01) and 90-day mortality rates of 2.6% (no GP) and 1.4% (GP, *p* = 0.03). Hence, one should be cautious concluding early follow-up visits reduce readmissions [22], when the event may occur before the visit. The lack of changes in the regression results upon excluding the new home nursing recipients from the analyses is potentially surprising, as newly receiving these services post-discharge could imply greater stroke severity compared to patients without home nursing post-discharge, leading to worse observed outcomes. However, this could be offset by a selection effect leading to better-observed outcomes relative to those not receiving home nursing, because patients dying shortly after discharge do not have time to switch to home nursing.

The variables used for risk adjustment had a substantial impact on the differences between care categories. Further validating our risk adjustment variables are predictors of readmission after stroke identified in a systematic review by Lichtman et al. [12] Those identified and available in our dataset were age, length of stay, incident stroke, comorbid conditions, discharge destination, previous hospitalizations and physical functioning. Additional support comes from Strowd et al., finding greater number of hospitalizations the year before stroke admission as a predictor of readmission [5]. Being one of the first studies to employ data linked from multiple registers covering an entire population with complete individual-level health service utilization histories that include hospitalizations, ambulatory care and demographic data is a significant strength. This allowed for individual-level analyses and risk adjustment with complete short- and long-term follow-up periods for all patients. The ability to account for all readmissions to any hospital regardless of original hospital adds additional strength.

Despite this study’s strengths, limitations are also present. The data are a few years dated, due to multiple reasons: considerable lag before inpatient register data are available for sampling and linkage, a slow application process and this being one of the first projects where municipal healthcare data are linked to national register data. Accomplishing individual level municipal health service data linkage to national registers requires establishing agreements with individual municipalities. Future research that is able to link national registries to service utilization data from multiple or all municipalities will broaden the geographic scope and generalizability of the findings. Home nursing was only divided into receiving the service or not. Readmission and mortality rates could differ based on the extent of home nursing received. We were unable to determine or account for the fact that not all readmissions are acute or wholly influenced by extenuating factors, rather planned or for elective procedures. Only analyzing acute readmissions and comparing the level and scope of the home nursing and rehabilitation provided would strengthen the results in future investigations. Patients discharged to facilities rather than home are often the most severe, but fall outside this study’s scope [37]. This caveat could limit the results’ overall generalizability to all stroke patients.

## Conclusion

Based on registry data from Oslo, stroke patients who receive home nursing after discharge perform similarly to patients without home nursing regarding mortality and short-term readmissions, after adjusting for health status and demographics at hospital admission. The post-discharge mortality rate for patients receiving rehabilitation was more than double that of patients not receiving rehabilitation, even after adjustment. No significant differences for early follow-up by GP on readmission or mortality were found when adjusting for health status and demographics at hospital admission. These results indicate that most of the differences in readmissions and mortality between groups receiving/not receiving home nursing or early follow-up are attributable to pre-stroke factors, while careful needs assessment at time of discharge as well as follow-up seem to be paramount for patients receiving post-discharge ambulatory rehabilitation services.

## Additional files

Additional file 1: **Table S1.** Multiple regression hazard ratios and 95% confidence intervals for the analysis of care categories. Hazard ratios and confidence intervals for all variables in multiple regression analyses of care categories. (DOCX 26 kb)

Additional file 2: **Table S2.** Multiple regression hazard ratios and 95% confidence intervals for the analysis of GP visits within 14 days. Description: Hazard ratios and confidence intervals for all variables in multiple regression analyses of GP visits within 14 days provided in Table S2 and Additional file 1: Table S1). (DOCX 26 kb)

## Abbreviations

ADL: Activities of daily living
CI: Confidence intervals
GP: General practitioner
HR: Hazard ratio
ICD-10: International Classification of Diseases 10th revision
IQR: Interquartile range
LOS: Length of stay
LTC: Long-term care
