# Supplementary material for: Comparisons of readmissions and mortality based on post-discharge ambulatory follow-up services received by stroke patients discharged home: a register-based study
Source: BMC Health Serv Res. 2019 Jan 5;19:4. doi: 10.1186/s12913-018-3809-z (PMC6321669; doi:10.1186/s12913-018-3809-z)
Supplement: Supplementary file 1 — Table S1. Multiple regression hazard ratios and 95% confidence intervals for the analysis of care categories. Hazard ratios and confidence intervals for all variables in multiple regression analyses of care categories. (DOCX 26 kb) [file 12913_2018_3809_MOESM1_ESM.docx]

**Table S1** Multiple regression hazard ratios and 95% confidence intervals for the analysis of care categories

|  | 90 days readmissions | | 365 days readmissions | | 90 days mortality | | 365 days mortality | |
| --- | --- | --- | --- | --- | --- | --- | --- | --- |
|  | HR | 95% CI | HR | 95% CI | HR | 95% CI | HR | 95% CI |
| No services | 1 |  | 1 |  | 1 |  | 1 |  |
| Rehabilitation | 1.45 | 1.06-1.97 | 1.80 | 1.45-2.22 | 2.69 | 1.80-4.03 | 2.38 | 1.83-3.09 |
| Home nursing | 1.08 | 0.74-1.57 | 1.31 | 1.02-1.66 | 0.81 | 0.45-1.47 | 1.00 | 0.71-1.41 |
| Home nursing and rehabilitation | 0.95 | 0.67-1.34 | 1.46 | 1.16-1.84 | 2.54 | 1.70-3.81 | 2.20 | 1.68-2.89 |
| Male | 1.21 | 0.96-1.53 | 1.20 | 1.03-1.41 | 1.27 | 0.96-1.7 | 1.30 | 1.08-1.57 |
| Age>90 years | 1 |  | 1 |  | 1 |  | 1 |  |
| Age 85-89 | 0.82 | 0.57-1.19 | 0.93 | 0.74-1.19 | 0.65 | 0.46-0.92 | 0.80 | 0.63-1.02 |
| Age 80-84 | 0.85 | 0.58-1.25 | 0.86 | 0.66-1.11 | 0.47 | 0.31-0.73 | 0.61 | 0.46-0.81 |
| Age 75-79 | 0.81 | 0.52-1.27 | 0.77 | 0.56-1.04 | 0.37 | 0.22-0.64 | 0.45 | 0.31-0.64 |
| Age 70-74 | 0.78 | 0.48-1.24 | 0.68 | 0.49-0.93 | 0.35 | 0.19-0.65 | 0.37 | 0.25-0.55 |
| Age 65-69 | 0.77 | 0.46-1.27 | 0.64 | 0.45-0.90 | 0.35 | 0.18-0.68 | 0.38 | 0.24-0.58 |
| Age 60-64 | 0.52 | 0.29-0.93 | 0.42 | 0.28-0.63 | 0.32 | 0.15-0.71 | 0.17 | 0.09-0.33 |
| Age 55-59 | 0.60 | 0.31-1.15 | 0.51 | 0.32-0.81 | 0.23 | 0.08-0.68 | 0.15 | 0.07-0.36 |
| Age 50-54 | 0.60 | 0.29-1.22 | 0.54 | 0.32-0.89 | 0.08 | 0.01-0.59 | 0.14 | 0.05-0.38 |
| Age<49 | 0.45 | 0.22-0.90 | 0.39 | 0.24-0.63 | 0.15 | 0.05-0.52 | 0.20 | 0.10-0.42 |
| Hypertension | 0.65 | 0.38-1.12 | 0.95 | 0.66-1.35 | 1.06 | 0.59-1.93 | 0.89 | 0.58-1.38 |
| Coronary artery disease | 1.29 | 0.81-2.04 | 1.52 | 1.12-2.07 | 0.88 | 0.50-1.57 | 0.86 | 0.57-1.28 |
| Atrial fibrillation | 0.62 | 0.35-1.10 | 0.92 | 0.64-1.31 | 1.33 | 0.76-2.32 | 1.25 | 0.84-1.87 |
| Cardiac insufficiency | 1.15 | 0.57-2.30 | 1.34 | 0.86-2.07 | 0.91 | 0.42-1.95 | 1.07 | 0.66-1.74 |
| Diabetes mellitus | 1.02 | 0.64-1.61 | 1.58 | 1.18-2.13 | 1.25 | 0.71-2.23 | 0.95 | 0.63-1.44 |
| COPD and asthma | 1.76 | 1.13-2.72 | 1.86 | 1.38-2.51 | 0.79 | 0.42-1.47 | 1.41 | 0.96-2.08 |
| Dementia | 1.38 | 0.90-2.13 | 1.04 | 0.76-1.42 | 0.90 | 0.57-1.43 | 1.13 | 0.84-1.52 |
| Renal insufficiency | 1.51 | 0.62-3.67 | 1.07 | 0.58-1.98 | 3.00 | 1.25-7.17 | 2.35 | 1.19-4.64 |
| LOS previous year=0 | 1 |  | 1 |  | 1 |  | 1 |  |
| LOS 1-10 days | 2.10 | 1.38-3.19 | 1.24 | 0.91-1.69 | 1.35 | 0.80-2.28 | 1.23 | 0.86-1.77 |
| LOS >10 days | 2.26 | 1.35-3.79 | 1.23 | 0.84-1.82 | 1.80 | 1.00-3.25 | 1.76 | 1.13-2.72 |
| ADL=0 | 1 |  | 1 |  | 1 |  | 1 |  |
| ADL 1-24 | 1.22 | 0.88-1.71 | 1.32 | 1.06-1.63 | 1.14 | 0.76-1.71 | 1.20 | 0.93-1.55 |
| ADL 25-39 | 1.66 | 1.21-2.28 | 1.47 | 1.19-1.83 | 1.48 | 1.04-2.09 | 1.34 | 1.05-1.70 |
| ADL >39 | 1.34 | 0.86-2.09 | 1.44 | 1.08-1.91 | 2.14 | 1.43-3.19 | 1.98 | 1.49-2.63 |
| Education primary | 1 |  | 1 |  | 1 |  | 1 |  |
| Secondary | 0.85 | 0.67-1.07 | 0.87 | 0.74-1.03 | 0.83 | 0.62-1.11 | 0.96 | 0.79-1.17 |
| Tertiary | 0.61 | 0.43-0.85 | 0.72 | 0.58-0.90 | 0.59 | 0.38-0.91 | 0.69 | 0.52-0.92 |
| Disability pension | 1.05 | 0.80-1.37 | 1.23 | 1.02-1.47 | 1.06 | 0.75-1.52 | 1.11 | 0.89-1.39 |
| Income <€21,000 | 1 |  | 1 |  | 1 |  | 1 |  |
| Income €21-31,000 | 1.03 | 0.78-1.37 | 0.96 | 0.80-1.17 | 0.91 | 0.66-1.26 | 0.89 | 0.71-1.10 |
| Income €31-41,000 | 0.95 | 0.69-1.32 | 0.95 | 0.76-1.18 | 0.82 | 0.55-1.23 | 0.90 | 0.70-1.17 |
| Income >€41,000 | 0.88 | 0.61-1.28 | 0.99 | 0.77-1.28 | 0.94 | 0.59-1.51 | 0.75 | 0.54-1.03 |
| Calendar year 2009 | 1 |  | 1 |  | 1 |  | 1 |  |
| 2010 | 0.95 | 0.65-1.38 | 0.97 | 0.77-1.24 | 0.96 | 0.65-1.43 | 1.02 | 0.78-1.33 |
| 2011 | 1.26 | 0.88-1.79 | 1.19 | 0.94-1.51 | 0.81 | 0.54-1.24 | 0.99 | 0.76-1.30 |
| 2012 | 1.12 | 0.79-1.61 | 1.03 | 0.82-1.31 | 0.63 | 0.41-0.96 | 0.94 | 0.72-1.23 |
| 2013 | 1.27 | 0.89-1.82 | 1.02 | 0.80-1.30 | 1.01 | 0.68-1.51 | 0.98 | 0.74-1.29 |
| 2014 | 1.12 | 0.77-1.62 | 1.16 | 0.89-1.52 | 0.60 | 0.37-0.96 | 0.64 | 0.45-0.93 |

HR=Hazard ratio

95% CI=95% confidence interval
