# Supplementary material for: Comparisons of readmissions and mortality based on post-discharge ambulatory follow-up services received by stroke patients discharged home: a register-based study
Source: BMC Health Serv Res. 2019 Jan 5;19:4. doi: 10.1186/s12913-018-3809-z (PMC6321669; doi:10.1186/s12913-018-3809-z)
Supplement: Supplementary file 2 — Table S2. Multiple regression hazard ratios and 95% confidence intervals for the analysis of GP visits within 14 days. Description: Hazard ratios and confidence intervals for all variables in multiple regression analyses of GP visits within 14 days provided in Table S2 and Additional file 1: Table S1). (DOCX 26 kb) [file 12913_2018_3809_MOESM2_ESM.docx]

**Table S2** Multiple regression hazard ratios and 95% confidence intervals for the analysis of GP visits within 14 days

|  | 14-90 days readmissions | | 14-365 days readmissions | | 14-90 days mortality | | 14-365 days mortality | |
| --- | --- | --- | --- | --- | --- | --- | --- | --- |
|  | HR | 95% CI | HR | 95% CI | HR | 95% CI | HR | 95% CI |
| GP within 14 days | 1.11 | 0.75-1.64 | 1.20 | 0.95-1.51 | 0.77 | 0.42-1.42 | 0.77 | 0.55-1.09 |
| Male | 0.87 | 0.60-1.25 | 1.02 | 0.81-1.28 | 1.81 | 0.98-3.35 | 1.34 | 0.96-1.86 |
| Age>90 years | 1.00 |  | 1.00 |  | 1.00 |  | 1.00 |  |
| Age 85-89 | 0.69 | 0.32-1.50 | 0.67 | 0.43-1.06 | 0.25 | 0.1-0.65 | 0.51 | 0.30-0.87 |
| Age 80-84 | 0.70 | 0.33-1.49 | 0.57 | 0.36-0.91 | 0.19 | 0.07-0.55 | 0.43 | 0.24-0.76 |
| Age 75-79 | 0.50 | 0.22-1.14 | 0.45 | 0.27-0.76 | 0.21 | 0.07-0.62 | 0.28 | 0.14-0.55 |
| Age 70-74 | 0.50 | 0.21-1.19 | 0.40 | 0.24-0.68 | 0.11 | 0.03-0.38 | 0.24 | 0.12-0.48 |
| Age 65-69 | 0.71 | 0.30-1.67 | 0.46 | 0.27-0.78 | 0.15 | 0.05-0.46 | 0.25 | 0.12-0.50 |
| Age 60-64 | 0.47 | 0.18-1.22 | 0.28 | 0.15-0.52 | 0.16 | 0.04-0.56 | 0.11 | 0.04-0.27 |
| Age 55-59 | 0.52 | 0.18-1.47 | 0.34 | 0.18-0.65 | 0.08 | 0.02-0.43 | 0.10 | 0.03-0.29 |
| Age 50-54 | 0.45 | 0.16-1.28 | 0.41 | 0.22-0.77 | 0.05 | 0.01-0.4 | 0.12 | 0.04-0.35 |
| Age<49 | 0.26 | 0.08-0.82 | 0.22 | 0.11-0.43 | 0.05 | 0.01-0.26 | 0.14 | 0.06-0.35 |
| Hypertension | 0.35 | 0.12-1.02 | 1.07 | 0.59-1.94 | 2.86 | 0.8-10.18 | 2.14 | 1.00-4.61 |
| Coronary artery disease | 1.31 | 0.57-3.03 | 1.32 | 0.78-2.23 | 0.35 | 0.09-1.42 | 0.51 | 0.22-1.18 |
| Atrial fibrillation | 1.15 | 0.50-2.64 | 1.29 | 0.76-2.19 | 0.20 | 0.02-2.37 | 1.01 | 0.44-2.29 |
| Cardiac insufficiency | 0.72 | 0.22-2.30 | 1.88 | 1.07-3.31 | 1.63 | 0.17-16.1 | 0.82 | 0.33-2.05 |
| Diabetes mellitus | 1.27 | 0.60-2.71 | 1.69 | 1.09-2.60 | 2.35 | 0.86-6.46 | 1.03 | 0.50-2.13 |
| COPD and asthma | 2.13 | 0.96-4.76 | 1.85 | 1.13-3.03 | 0.61 | 0.23-1.61 | 2.05 | 1.13-3.73 |
| Dementia | 1.31 | 0.42-4.12 | 1.27 | 0.62-2.60 | 0.41 | 0.04-3.91 | 1.39 | 0.68-2.85 |
| Renal insufficiency | 1.78 | 0.32-9.74 | 1.10 | 0.43-2.83 | 1.09 | 0.1-11.87 | 0.67 | 0.15-3.02 |
| LOS previous year=0 | 1.00 |  | 1.00 |  | 1.00 |  | 1.00 |  |
| LOS 1-10 days | 2.97 | 1.49-5.90 | 1.41 | 0.88-2.27 | 1.63 | 0.41-6.42 | 1.13 | 0.52-2.45 |
| LOS >10 days | 3.23 | 1.22-8.54 | 1.65 | 0.90-3.02 | 4.59 | 1.40-15.01 | 3.48 | 1.58-7.64 |
| ADL=0 | 1.00 |  | 1.00 |  | 1.00 |  | 1.00 |  |
| ADL 1-24 | 1.40 | 0.80-2.44 | 1.40 | 1.00-1.96 | 0.38 | 0.10-1.42 | 0.96 | 0.57-1.62 |
| ADL 25-39 | 1.59 | 0.89-2.84 | 1.73 | 1.21-2.48 | 1.22 | 0.49-3.03 | 1.23 | 0.75-1.99 |
| ADL >39 | 1.80 | 0.81-4.02 | 3.08 | 1.97-4.82 | 1.05 | 0.29-3.83 | 2.83 | 1.60-5.01 |
| Education primary | 1.00 |  | 1.00 |  | 1.00 |  | 1.00 |  |
| Secondary | 0.87 | 0.59-1.29 | 1.04 | 0.80-1.35 | 1.58 | 0.80-3.12 | 1.03 | 0.72-1.48 |
| Tertiary | 0.51 | 0.29-0.88 | 0.81 | 0.58-1.14 | 1.16 | 0.47-2.83 | 0.63 | 0.39-1.02 |
| Disability pension | 1.12 | 0.76-1.67 | 1.60 | 1.24-2.05 | 0.59 | 0.23-1.54 | 0.95 | 0.62-1.45 |
| Income <€21,000 | 1.00 |  | 1.00 |  | 1.00 |  | 1.00 |  |
| Income €21-31,000 | 0.85 | 0.53-1.37 | 0.82 | 0.61-1.10 | 0.87 | 0.41-1.85 | 0.70 | 0.46-1.05 |
| Income €31-41,000 | 0.77 | 0.45-1.31 | 0.86 | 0.62-1.19 | 0.40 | 0.16-1.03 | 0.69 | 0.44-1.09 |
| Income >€41,000 | 1.06 | 0.60-1.86 | 0.96 | 0.67-1.38 | 0.91 | 0.37-2.28 | 0.82 | 0.49-1.39 |
| Calendar year 2009 | 1.00 |  | 1.00 |  | 1.00 |  | 1.00 |  |
| 2010 | 0.95 | 0.52-1.74 | 0.88 | 0.61-1.27 | 1.19 | 0.56-2.52 | 1.12 | 0.69-1.82 |
| 2011 | 1.30 | 0.75-2.25 | 1.36 | 0.98-1.88 | 0.34 | 0.13-0.90 | 1.06 | 0.67-1.69 |
| 2012 | 1.42 | 0.81-2.49 | 1.09 | 0.76-1.55 | 0.45 | 0.18-1.18 | 1.32 | 0.83-2.12 |
| 2013 | 1.24 | 0.70-2.20 | 0.87 | 0.59-1.26 | 0.55 | 0.23-1.34 | 0.97 | 0.58-1.60 |
| 2014 | 0.81 | 0.41-1.59 | 0.98 | 0.63-1.55 | 0.46 | 0.16-1.35 | 0.44 | 0.19-1.01 |

GP=General practitioner

HR=Hazard ratio

95% CI=95% confidence interval
